# Response to apatinib and camrelizumab combined treatment in a radioiodine refractory differentiated thyroid cancer patient resistant to prior anti-angiogenic therapy: A case report and literature review

**DOI:** 10.3389/fimmu.2022.943916

**Published:** 2022-08-08

**Authors:** Jiayi Li, Xin Zhang, Zhuanzhuan Mu, Di Sun, Yuqing Sun, Yansong Lin

**Affiliations:** ^1^ Department of Nuclear Medicine, Peking Union Medical College Hospital, Beijing, China; ^2^ Beijing Key Laboratory of Molecular Targeted Diagnosis and Therapy in Nuclear Medicine, Beijing, China; ^3^ State Key Laboratory of Complex Severe and Rare Diseases, Peking Union Medical College Hospital, Chinese Academy of Medical Science and Peking Union Medical College, Beijing, China; ^4^ Chinese Academy of Medical Sciences & Peking Union Medical College, Beijing, China

**Keywords:** radioactive iodine refractory, differentiated thyroid cancer, combination immunotherapy, multitargeted kinase inhibitors plus PD-1/PD-L1 inhibitors therapy, apatinib plus camrelizumab treatment

## Abstract

**Background:**

Patients with radioactive iodine refractory progressive (RAIR) differentiated thyroid cancer (DTC) often developed resistance after first-line therapy. Apatinib plus camrelizumab is a therapy with promising efficacy in patients with other malignant cancers. Herein, we presented a case of progressive RAIR DTC treated with apatinib plus camrelizumab.

**Case presentation:**

We reported a 43-year-old man diagnosed as DTC with metastases in the lungs, the 7th cervical vertebra, and malignant lymph nodes mainly in the mediastinum. While initially showing disease stabilization after giving the first-line multitargeted kinase inhibitor (MKI) therapy, the patient developed progressive disease and was enrolled into a combined therapy with both apatinib and camrelizumab on November 10, 2020. Upon the first 6 months, the combination therapy showed disease control in terms of both stable structural lesions and biochemical thyroglobulin (Tg) level. Six months later, a decrease over the targeted lesions was observed and a partial response (PR) according to RECIST 1.1 criteria was finally achieved upon 12 months’ assessment, followed by the decline in serum Tg level. The main adverse event was occasional diarrhea without treatment interruption.

**Conclusion:**

We reported a case with RAIR DTC that benefited from combination immunotherapy, apatinib plus camrelizumab, after resistance from donafenib. We observed a gradually getting better efficacy and a mild and long duration of this combination therapy and hoped to provide a therapeutic choice for these patients.

## Introduction

The progression in differentiated thyroid cancer (DTC) can occur in up to approximately 20% after standard therapeutic approaches in the 10-year follow-up (1). After radioactive iodine (RAI) therapy, loss of the ability of iodine uptake can occur in two-thirds of these patients, which are called RAI-refractory, progressive differentiated thyroid cancer (RAIR-DTC) (2). Those with RAIR-DTC have a 10-year survival rate less than 10% survival, which greatly reduce clinical dilemma.

Currently, two multitargeted kinase inhibitors (MKI) sorafenib and lenvatinib have been approved for use in patients with progressive RAIR DTC by the National Medical Products Administration (NMPA) as the first-line systemic therapy based upon their promising antitumor activity (3). However, most RAIR-DTC patients developed resistance to MKIs over the following 1 to 2 years (4). In addition, drug-induced adverse effects were commonly seen with MKI treatment under standard doses, which may downgrade the patients’ quality of life and even lead to termination of MKI therapy (5). Cabozantinib is a recently approved therapy after first-line resistance (6). However, it is still a MKI therapy and could not be available in Chinese patients.

Immunotherapy PD-1/PD-L1 inhibitors have also achieved promising results in many tumor types such as melanoma and non-small cell lung cancer (7). A non-randomized, phase Ib trial KEYNOTE-028 estimated response of patients with RAIR-DTC to PD-1 and observed objective responses in a minority of patients (8). The influence of PD-1 treatment to RAIR-DTC must be substantiated in subsequent clinical trials. The combination of immunotherapy and MKIs is a topic of high interest in the treatment of advanced malignant tumors. Lenvatinib plus pembrolizumab showed more potent antitumor activity compared with either agent alone in mouse xenograft (9) and human studies (10), showing promising benefit in RAIR-DTC (11). Apatinib is a domestic MKI which showed rapid and significant efficacy in its phase II and phase III studies conducted in progressive RAIR-DTC (12), and apatinib plus camrelizumab is a combined therapy which has shown promising efficacy recently in patients with hepatocellular carcinoma or neuroendocrine carcinoma (13).

To our knowledge, apatinib combined with camrelizumab therapy has not been systematically reported in thyroid cancers. We presented here a case of progressive RAIR-DTC treated with apatinib plus camrelizumab (ClinicalTrials.gov Identifier: NCT04560127).

## Case presentation

A 43-year-old man diagnosed with follicular thyroid cancer (FTC) with metastases in the lungs, the 7th cervical vertebra, and malignant lymph nodes mainly in the mediastinum and resistance to first-line MKI was given a combined therapy of antiangiogenic MKI apatinib and anti-PD-1 antibody camrelizumab in October 2020.

**Figure 1:**
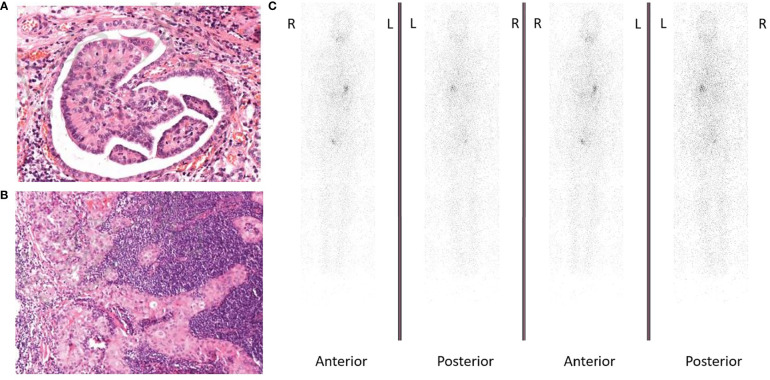
**(A)** Hematoxylin and eosin (H&E) staining showing follicular thyroid cancer cells (magnification ×100). **(B)** Hematoxylin and eosin (H&E) staining showing lymph node metastasis from follicular thyroid cancer (magnification ×100). **(C)** Whole-body iodine scan showed radioactive iodine refractory.

In 2015, the patient was diagnosed with DTC (**Figures 1A**, **B**) and received radical thyroidectomy and subsequent RAI therapy. Mutation analysis was performed, and results were positive for TERT, BRAF p.L597Q, and VEGF, while negative for RAS and BRAF p.VAL600. During the regular follow-up including cervical ultrasound and chest computed tomography (CT) scanning accompanied by TSH suppression therapy, recurrences were found in cervical lymph nodes, mediastinal lymph nodes, lungs, and the 7th cervical vertebra in 2017. Therefore, he received cervical lymph node dissection and several subsequent RAI therapies. However, his pulmonary lesions did not take iodine revealed by whole-body iodine scan after last RAI therapy in October 2019 (**Figure 1C**). Thus, he was identified as RAIR after receiving several surgeries and RAI therapies with a cumulative dose of 670 mCi. He was then given a first-line MKI in October 2019. While initially showing disease stabilization after giving the first-line MKI, the patient developed progressive disease (PD) and terminated the first-line MKI therapy on September 10, 2020.

**Figure 2:**
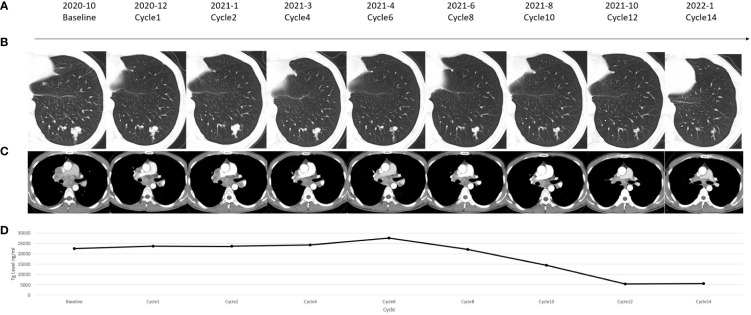
**(A)** Timeline of the reported case. **(B)** Chest computed tomography (CT) scanning showed change of a pulmonary lesion which was located in the left lower lobe of the lung. **(C)** Chest CT scanning showed change of a subcarinal lymph node lesion. **(D)** Change in the serum thyroglobulin (Tg) level in each assessment.

Followed by a 4-week discontinuation, the patient was enrolled into an exploratory phase II clinical trial combining antiangiogenic MKI apatinib and anti-PD-1 antibody camrelizumab in November 2020. The therapeutic schedule was given by 250 mg apatinib orally once daily and 200 mg camrelizumab intravenously once every 2 weeks in a 4-week cycle. The patient was evaluated every cycle in the first 2 cycles and every 2 cycles thereafter. During the first 6 months’ assessment, the combination therapy initially showed disease control in terms of both stable structural lesions and biochemical thyroglobulin (Tg) level (**Figure 2**). Six months later from April 2021, the targeted and non-targeted lesions began to shrink rapidly followed by a rapid decrease in serum Tg, and a partial response (PR) according to RECIST 1.1 criteria was finally achieved upon 12 months’ assessment in October 2021 (targeted lesions: 4.7 to 2.9 cm) (**Figures 2B**, **C**). Along with this process, the biochemical Tg level also decreased from 22,481 ng/ml (October 2020) to 5,351 ng/ml (October 2021) (**Figure 2D**).

During this process, the main adverse event was occasional diarrhea without treatment interruption. The patient was in the state of PR at the last assessment and will be followed up in the future.

## Discussion

We presented here a case of RAIR DTC with disease progression that responded to apatinib plus camrelizumab after resistance to an antiangiogenic drug. To our knowledge, we believed this was the first case of RAIR DTC with disease progression treated with apatinib plus camrelizumab. The patient showed a durable and safe response of nearly 1 year.

In our case, the patient with RAIR DTC got resistant to the first MKI donafenib. There were no public-approved treatments for RAIR DTC patients who became resistant to MKI in China. A number of previous clinical studies have explored the use of camrelizumab combined with apatinib for hepatocellular carcinoma, NSCLC, SCLC, cervical cancer, breast cancer, osteosarcoma, esophageal squamous cell carcinoma, colorectal carcinoma, gestational trophoblastic neoplasia, biliary tract cancer, and gastric and esophagogastric junction cancer and showed promising results (14–25) (**Table 1**). Another combination therapy of immunotherapy PD-1 inhibitors and MKIs, lenvatinib plus pembrolizumab, showed disease control in thyroid cancer. Luongo et al. (26) showed that patients with paucicellular variant anaplastic thyroid cancer reached PR 5 months after giving lenvatinib plus pembrolizumab and PR persisted over 18 months. Dierks et al. (27) and Iyer et al. (28) showed that disease control happened in over half of patients with thyroid cancer after lenvatinib plus pembrolizumab therapy (**Table 2**). The efficacy of these studies was consistent with our research. The mechanism may be related to that use of MKI could normalize the abnormal tumor vasculature, thus increasing the infiltration of immune effector cells like CD4+ and CD8+ T cells into tumors (30–32). Preclinical experiments also found that apatinib could enhance the efficacy of PD-1/PD-L1 blockade through alleviating hypoxia, increasing the infiltration of CD8+ T cells, and reducing tumor-associated macrophage recruitment and TGFβ amounts in both tumor and serum (33). The selection of dosing is also a question needed to be resolved. Previous fundamental studies observed a more favorable microenvironment for immunotherapy in low-dose apatinib instead of high-dose treatment (33). Therefore, the dose of our case is 250 mg apatinib in combination with 200 mg camrelizumab, which was consistent with previous studies (**Table 1**).

**Table 1:** Clinical trials for camrelizumab combined with apatinib for the treatment of other diseases.

Author	Disease	Interventions	Efficacy	Adverse event
Jianming Xu et al. (15)	Advanced hepatocellular carcinoma	Camrelizumab 200 mg (for bodyweight ≥50 kg) or 3 mg/kg (for bodyweight <50 kg) every 2 weeks plus apatinib 250 mg daily	First-line ORR 34.3%, median PFS 5.7 months, 12-month survival rate 74.7%; second-line ORR 22.5%, median PFS 5.5 months, 12-month survival rate 68.2%	Grade ≥3 AEs 77.4%, most hypertension 34.2%; serious AEs 28.9%, treatment-related deaths 1.1%
Kuimin Mei et al. (14)	Advanced primary liver cancer	Camrelizumab (3 mg/kg, once every 2 weeks) plus apatinib (125, 250, 375, or 500 mg; once per day)	ORR 10.7%, median PFS 3.7 months, median OS 13.2 months	grade ≥3 AEs 92.9%
Shengxiang Ren et al. (16)	Advanced non-squamous NSCLC	Camrelizumab 200 mg every 2 weeks plus apatinib 250 mg once daily	ORR 40.0%, disease control rate 92.0%, median PFS 9.6 months	Grade ≥3 AEs 80%
Yun Fan et al. (17)	Extensive-stage SCLC	Camrelizumab 200 mg every 2 weeks plus apatinib 375 mg once daily	ORR 34.0%, median PFS 3.6 months, median OS 8.4 months	Grade ≥3 AEs 72.9%; discontinued 8.5%
Chunyan Lan et al. (18)	Advanced cervical cancer	Camrelizumab 200 mg every 2 weeks and apatinib 250 mg once per day	ORR 55.6%, median PFS 8.8 months	Grade ≥3 AEs 71.1%, hypertension 24.4%, anemia 20.0%, fatigue 15.6%
Jieqiong Liu et al. (19)	Advanced triple-negative breast cancer	Camrelizumab every 2 weeks with apatinib 250 mg at either continuous dosing (d1–d14) or intermittent dosing (d1–d7)	Continuous cohort: ORR 43.3%, median PFS 3.7 months; intermittent cohort ORR 0, median PFS 1.9 months	Grade ≥3 AEs 46.7%
Lu Xie et al. (20)	Advanced osteosarcoma	Apatinib 500 mg once daily plus camrelizumab 200 mg by every 2 weeks	ORR 20.9%, Median PFS 6.2 months, median OS 11.3 months	Grade ≥3 AEs 69.8%
Xiangrui Meng et al. (21)	Advanced esophageal squamous cell carcinoma	Camrelizumab 200 mg once every 2 weeks plus apatinib 250 mg once daily	ORR 34.6%, disease control 78.8%, median PFS 6.8 months, median OS 15·8 months	Grade ≥3 AEs 44%; most increased aspartate aminotransferase 19%, increased gamma-glutamyl transferase 19%, increased alanine aminotransferase 10%
Chao Ren et al. (22)	Metastatic colorectal cancer	Camrelizumab 200 mg every 2 weeks and apatinib 250–375 mg once daily	ORR 0%, disease control rate 22.2%, median PFS 1.83 months, median OS 7.80 months	Grade 3 AEs 90%, most hypertension 30%
Hongyan Cheng et al. (23)	Chemorefractory or relapsed gestational trophoblastic neoplasia	Camrelizumab 200 mg every 2 weeks plus apatinib 250 mg once per day	ORR 55%, complete response 50%	Grade 3 AEs 60%
Dongxu Wang et al. (24)	Advanced biliary tract cancer	Apatinib at 250 mg per a day and camrelizumab intravenously at 200 mg every three weeks	PR 19%, disease control rate 71.4%, median PFS 4.4 months, median OS 13.1 months	Grade ≥3 AEs 63.6%
Jianming Xu et al. (25)	Advanced hepatocellular carcinoma, gastric, or esophagogastric junction cancer	Camrelizumab 200 mg every 2 weeks and apatinib 125–500 mg once daily	PR 50.0%, ORR 30.8%	Grade ≥3 AEs 60.6%

ORR, objective response rate; PFS, progression-free survival; OS, overall survival; AEs, adverse effects; PR, partial response. SD, stable disease; PD, progressive disease.

**Table 2:** Clinical studies for lenvatinib combined with pembrolizumab for the treatment of thyroid cancer.

Author	Disease	Interventions	Efficacy	Adverse event
Christine Dierks et al. (27)	6 patients with metastatic ATC and 2 patients with PDTC	Lenvatinib 14–24 mg daily combined with pembrolizumab 200 mg every 3 weeks	Overall PR 6/8, ATCs ORR 66%, median PFS 17.75 months, median OS 18.5 months	Grade ≥3 AEs 50%
Cristina Luongo et al. (26)	One patient with paucicellular variant ATC	Lenvatinib (24 mg daily) in combination with pembrolizumab (200 mg every 21 days)	PR achieved after 5 months, PR of lung metastasis persisted over 18 months	Grade 3 diarrhea, vomiting, and weight loss
Priyanka C Iyer et al. (28)	12 patients with ATC	6 patients: pembrolizumab plus dabrafenib and trametinib; 5 patients: pembrolizumab plus lenvatinib; 1 patient: pembrolizumab plus trametinib	PR 5/12 (42%), SD 4/12 (33%), PD 3/12 (25%), median OS 6.93 months, median PFS 2.96 months	Fatigue, anemia, hypertension

ATC, anaplastic thyroid carcinoma; PDTC, poorly differentiated thyroid carcinoma; ORR, objective response rate; PFS, progression-free survival; OS, overall survival; AEs, adverse effects; PR, partial response; SD, stable disease; PD, progressive disease.

The response pattern of this combined therapy featured as initial stabilization and delayed tumor reduction was quite different from the current effect of mono-MKI therapy, which may suggest the difference between our combined effect and monotherapy. As we previously mentioned, patients always showed rapid response to antiangiogenic drugs but progressed eventually. Previous studies found that in apatinib monotherapy, the serum Tg level decreased as early as 2 weeks (34), the median time to objective response was 1.9 months (29), and an expected time to progression was between 11 and 18 months (33, 35). Meanwhile, the response to immunotherapy seems to be more durable. A study found that time to response for patients with DTC after PD-1 inhibitor therapy who reached PR was 4 to 5 months (11). For our patient, time to PR was much longer than previous research of apatinib monotherapy. In addition, we did not find the patient who showed the same response pattern in apatinib monotherapy. Therefore, the first 6-month response in this patient may suggest the effect of a low-dose MKI to some extent, while the subsequent response may suggest the effect of immunotherapy. The long duration indicated a mild and lasting therapeutic effect of low-dose apatinib combined with camrelizumab in thyroid cancer.

During this process, the main adverse event was occasional diarrhea without treatment interruption. Adverse events including hand–foot syndrome, hypertension, and proteinuria are commonly observed in apatinib therapy but did not appear in this patient (36), which may suggest the safety of a low-dose schedule. The reactive cutaneous capillary endothelial proliferation was a common adverse event in camrelizumab therapy (37). There is a hypothesis that apatinib, a kind of antiangiogenic drug, would inhibit the proliferation of endothelial cells and thus would present a counteracting effect against adverse effects of camrelizumab. Compared with camrelizumab monotherapy, the skin capillary hyperplasia symptoms in combined therapy were lower than before (**Table 1**).

The change in serum Tg level in our case also suggested the potential prediction value of serum Tg on target and immunotherapy. The correlation between ps-Tg and therapeutic response was proposed in 2015 American Thyroid Association guidelines, and the relationship between Tg and apatinib plus camrelizumab therapy needed to be confirmed (2). Therefore, our results indicated the value of Tg in observing response to target therapy.

## Data availability statement

The original contributions presented in the study are included in the article/supplementary material. Further inquiries can be directed to the corresponding author.

## Ethics statement

The study was approved by the Ethics Committee of Peking Union Medical College Hospital. The patients/participants provided their written informed consent to participate in this study.

## Author contributions

JL and XZ: conceptualization; data curation; formal analysis; investigation; methodology; software; visualization; writing—original draft. ZM, DS, and YS: supervision; validation; visualization; writing—review and editing. YL: conceptualization; funding acquisition; methodology; project administration; resources; software; supervision; validation; visualization; writing—review and editing. All authors contributed to the article and approved the submitted version.

## Funding

This study was funded by the CAMS Innovation Fund for Medical Sciences (CIFMS) (No. 2020-I2M-2-003), the CSCO-Hengrui Research Foundation (No. Y-HR2018-143, Y-HR2018-144), the National Natural Science Foundation of China (grant 81771875), and the Project on Inter-Governmental International Scientific and Technological Innovation Cooperation in the National Key Projects of Research and Development Plan (grant 2019YFE0106400). In addition, apatinib and camrelizumab were provided by Jiangsu Hengrui Pharmaceuticals Co Ltd.

## Conflict of interest

The authors declare that this study was funded by the CAMS Innovation Fund for Medical Sciences (CIFMS) (No. 2020-I2M-2-003), the CSCO-Hengrui Research Foundation (No. Y-HR2018-143, Y-HR2018-144), the National Natural Science Foundation of China (grant 81771875), the Project on Inter-Governmental International Scientific and Technological Innovation Cooperation in the National Key Projects of Research and Development Plan (grant 2019YFE0106400). In addition, apatinib and camrelizumab were provided by Jiangsu Hengrui Pharmaceuticals Co Ltd. The funder was not involved in the study design, collection, analysis, interpretation of data, the writing of this article or the decision to submit it for publication.

## Publisher’s note

All claims expressed in this article are solely those of the authors and do not necessarily represent those of their affiliated organizations, or those of the publisher, the editors and the reviewers. Any product that may be evaluated in this article, or claim that may be made by its manufacturer, is not guaranteed or endorsed by the publisher.
